# Time course of soluble P-selectin and von Willebrand factor levels in trauma patients: a prospective observational study

**DOI:** 10.1186/1757-7241-21-70

**Published:** 2013-09-14

**Authors:** Ning Tang, Shiyu Yin, Ziyong Sun, Yingying Pan

**Affiliations:** 1 Tongji Hospital, Tongji Medical College, Huazhong University of Science and Technology, Wuhan, Hubei 430030, China

**Keywords:** Soluble P-selectin, von Willebrand factor, Trauma, Trauma-induced coagulopathy

## Abstract

**Background:**

Coagulopathy often develops in patients with serious trauma and is correlated with the clinical outcome. The contribution of platelet activity and endothelial dysfunction to trauma-induced coagulopathy remain to be defined. The purpose of this study was to investigate the time courses of soluble P-selectin (sPsel, an index of platelet activation) and von Willebrand factor (VWF, an index of endothelial dysfunction) in trauma patients and elucidate their relationship to coagulation parameter levels, the presence of coagulopathy, and patient outcome.

**Methods:**

This prospective observational study, which took place in a university hospital intensive care unit (ICU), included 82 severely injured trauma patients. The sPsel, VWF antigen, protein C, and factor VII levels were measured and routine coagulation tests were performed upon admission to ICU and daily within the first week. The 30-day mortality rate was also determined.

**Results:**

Thirty-seven (45.1%) patients developed coagulopathy upon admission to the ICU, and the 30-day mortality rate was 20.7% (n = 17). Both the admission sPsel and VWF levels were lower in patients with coagulopathy than in those without (*p* < 0.05) and were significantly correlated with the protein C and factor VII levels, respectively (all *p* < 0.05). The VWF levels were lower during the first 3 days and higher on day 7 after admission in nonsurvivors than in survivors (all *p* < 0.05). No significant differences in sPsel levels were found between nonsurvivors and survivors on each day during the first week.

**Conclusion:**

In severely injured trauma patients in the ICU, lower levels of sPsel and VWF on admission were associated with the presence of coagulopathy and might not predict a better outcome. An increase in the VWF level at the end of the first week after admission to ICU was associated with increased 30-day mortality.

## Background

When death due to exsanguination does not immediately occur in trauma patients, bleeding and shock increase the risk of multiple organ failure and late mortality. Much attention has been given to coagulopathy because it is an independent predictor of mortality. The proposed mechanism of trauma-induced coagulopathy (TIC) is that shock and tissue injury lead to systemic anticoagulation by widespread endothelial damage and activation of protein C (PC) [1]. Thrombin generation, PC activation, and hyperfibrinolysis are all associated with endothelial glycocalyx degradation [2]. Meanwhile, TIC and the combined effects of shock and hypothermia would theoretically result in platelet dysfunction through disruption of activation and adhesion pathways [3]. To date, however, the contribution of platelet activity and endothelial damage/dysfunction to TIC remain to be defined; doing so may offer novel therapeutic targets [4].

Soluble P-selectin (sPsel) and von Willebrand factor (VWF) have been described as plasma markers of platelet activation and endothelial damage/dysfunction, respectively [5,6]. Characterizing the evolution of sPsel and VWF levels in trauma patients may help to understand the roles of platelet activation and endothelial dysfunction in trauma and TIC. Our hypothesis was that in severe trauma patients, the sPsel and VWF levels on admission are associated with TIC and that changes in these levels in the early stage may predict outcomes. Therefore, the aim of our study was to investigate whether correlations exist among levels of sPsel, VWF, and coagulation parameters; the presence of coagulopathy; and 30-day mortality among trauma patients.

## Methods

All consecutive trauma patients admitted to the intensive care unit (ICU) between May and September 2012 and who had a >48-h estimated length of stay in the ICU were included in this study. Exclusion criteria were as follows: <18 y of age, pregnant, or currently undergoing anticoagulation or antiplatelet therapy.

This study was approved by the Ethics Committee of Tongji Hospital (Wuhan, China), and written informed consent was obtained from all patients or their family members.

On admission to the emergency room, patients were initially evaluated, were resuscitated (with crystalloids), and then underwent emergency surgery when necessary. Only the patients transferred to the ICU within 24 h of the injury were included in the study. Blood samples were collected daily after admission to ICU for 1 week; the first sampling was completed as early as possible following admission. The Injury Severity Score (ISS) was also obtained on admission to the ICU.

In the central clinical laboratory of Tongji Hospital, the blood samples were collected into citrate vacuum tubes and centrifuged at 2000 × *g* for 10 min. Platelet-poor plasma was obtained for immediate analysis or storage at −70°C within 1 month. sPsel concentrations were determined by commercially available ELISA kits (R&D Systems, Inc., USA). VWF antigen, coagulation factor VII (FVII), and PC concentrations were determined and routine coagulation tests (prothrombin time, activated partial thromboplastin time, and fibrinogen) were performed using the STA-R automated coagulation analyzer and commercially available reagents (Diagnostica Stago, France). Data on hemoglobin (Sysmex 2100; Sysmex Corp., Japan) and blood glucose (Modular DPP; Roche Corp., USA) on the first day were collected from the laboratory information system.

TIC was defined as a prothrombin ratio (PTR) of >1.2 within 1 day after admission according to a recent epidemiological study of >5000 patients [7].

### Statistical analysis

The Kolmogorov–Smirnov test was used to verify the normality of distribution of continuous variables. Normally distributed quantitative variable were compared between patients with and without coagulopathy and between survivors and nonsurvivors by Student’s t test and described as mean ± standard deviation. Non-normally distributed quantitative variables were compared by the Mann–Whitney U test and described as median values and interquartile ranges; qualitative variables were compared by the chi-square test and described in figures and as percentages. Pearson’s or Spearman’s correlation coefficients were calculated to determine correlations between sPsel/VWF levels and other variables. A *p* value of <0.05 was considered to be statistically significant. Data were analyzed using SPSS 13.0 for Windows (SPSS Inc., Chicago, IL).

## Results

A total of 82 patients (54 male, 28 female; mean age, 45.3 years) were enrolled in this study. No antithrombin, recombinant activated human PC, or recombinant FVII concentrations were given for the included patients. None of the patients had received blood product transfusions until arrival at the hospital. Thirty-seven (45.1%) patients developed coagulopathy upon admission to the ICU or during the first day. The 30-day mortality rate was 20.7% (n = 17); 13 patients died of multiple organ dysfunction syndrome, 3 died of respiratory dysfunction, and 1 died of acute cerebral hemorrhage. Table 1 delineates the demographics, clinical characteristics, and administration of blood components on the first day stratified into patients with and without coagulopathy.

**Table 1:** Patients stratified by presence of coagulopathy

	**Total**	**Coagulopathy**	**Non-coagulopathy**	***P*value**
	**(n = 82)**	**(n = 37)**	**(n = 45)**	
Age (y)	45.3 ± 8.2	47.2 ± 7.1	41.5 ± 5.9	0.419
Male, n (%)	54 (65.9%)	26 (70.3%)	28 (62.2%)	0.490
Blunt trauma	79 (96.3%)	35 (94.6%)	44 (97.8%)	0.550
Type of trauma				
Cerebral	18	8	10	
Extremities	2	0	2	
Abdominal	6	1	5	
Thoracic	10	3	7	
Multi trauma	43	24	19	
Other	3	1	2	
ISS	27.6 ± 2.2	33.8 ± 3.5	23.3 ± 1.8	0.003
30-day mortality, n (%)	17 (20.7%)	13 (35.1%)	4 (8.9%)	0.004
Laboratory analyses on admission				
sPsel (NR: 20–44 ng/mL)	54.8 ± 22.6	49.4 ± 26.0	59.5 ± 18.2	0.044
VWF (NR: 66–176%)	224.1 (198.3-245.1)	205.8 (135.5-231.1)	244.2 (199.2-247.2)	0.014
PTR (NR: 0.88-1.12)	1.18 (1.07-1.56)	1.68 (1.41-1.74)	1.08 (1.05-1.13)	<0.001
Protein C (NR: 70–130%)	76.7 ± 38.8	48.0 ± 19.1	101.5 ± 34.2	<0.001
Factor VII (NR: 55–170%)	74.4 ± 37.0	46.0 ± 24.2	98.9 ± 27.5	<0.001
Hemoglobin (NR: 110-160 g/L)	101.1 ± 29.9	90.3 ± 29.4	109.8 ± 27.6	0.022
Glucose	8.39 ± 2.78	8.86 ± 3.15	8.09 ± 2.38	0.411
(NR: 4.11-6.05 mmol/L)				
Transfusions in units for the first day				
PLTC	1.5 (1.0-2.0)	1.5 (1.0-2.0)	0	
	(n = 5)	(n = 5)		
FFP	3.5 (2.5-7.0)	4.0 (3.0-7.5)	2 and 4 units	0.230
	(n = 9)	(n = 7)	(n = 2)	
PRBC	2.5 (1.5-4.0)	2 and 2 units	2.5 (1–4.5)	0.315
	(n = 13)	(n = 2)	(n = 11)	

Frequencies are given as count with percentage and were compared by the chi-square test. Transfusions (units) and admission VWF and PTR levels are given as medians (interquartile range) and were compared by the Mann–Whitney U test. Levels of other parameters, age, and ISS are given as means ± standard deviations and were compared by Student’s t test. *ISS* Injury Severity Score, *NR* normal range, *PLTC* platelet concentrates, *FFP* fresh frozen plasma, *PRBC* packed red blood cells.

**Figure 1:**
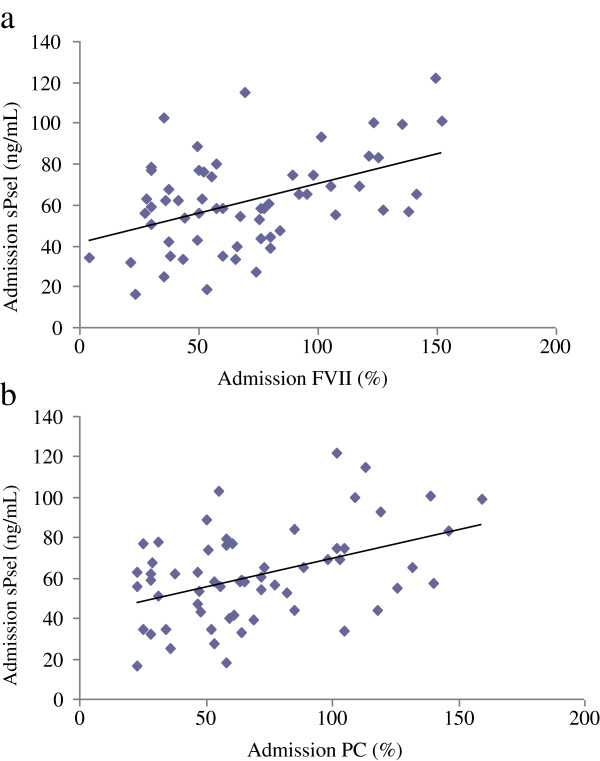
**Correlations between admission levels of sPsel and coagulation parameters.** Correlations between admission levels of sPsel and FVII **(a)** and PC **(b)**. Abbreviations: sPsel, soluble P-selectin; FVII, coagulation factor VII; PC, protein C.

**Figure 2:**
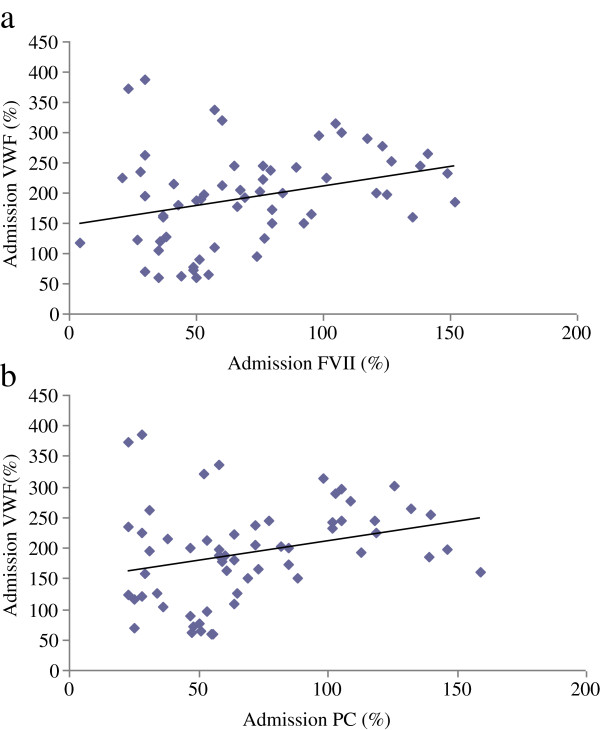
**Correlations between admission levels of VWF and coagulation parameters.** Correlations between admission levels of VWF and FVII **(a)** and PC **(b)**. Abbreviations: VWF, von Willebrand factor; FVII, coagulation factor VII; PC, protein C.

Correlations between levels of sPsel/VWF and levels of coagulation parameters (PC and FVII) upon admission to the ICU are presented in Table 2; significant associations were noted (Figures 1 and 2).

**Table 2:** Correlations between levels of sPsel/VWF and coagulation parameters upon admission

	**Admission sPsel**		**Admission VWF**	
	**Pearson’s correlation coefficient**	***p *value**	**Spearman’s correlation coefficient**	***p *value**
Admission PC	0.401	<0.001	0.245	0.026
Admission FVII	0.456	<0.001	0.253	0.022

*sPsel* soluble P-selectin, *VWF* von Willebrand factor, *PC* protein C, *FVII* coagulation factor VII.

**Figure 3:**
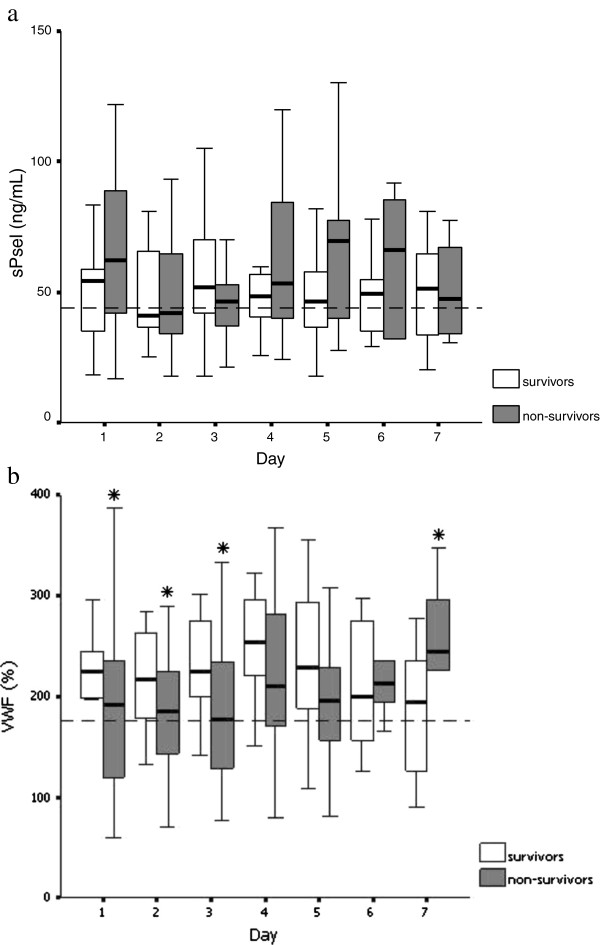
**Evolution of sPsel and VWF levels during the first week after admission according to outcome. (a)** Evolution of sPsel levels (ng/ml) during the first week after admission according to outcome. The dashed line represents the upper limit of normal for sPsel levels (44 ng/ml). No significant differences were found in sPsel levels on each day between survivors and nonsurvivors (by Student’s t test or the Mann–Whitney U test). **(b)** Evolution of VWF levels (%) during the first week after admission according to outcome. The dashed line represents the upper limit of normal for VWF levels (176.0%). *p < 0.05 compared with survivors (Mann–Whitney U test).

The evolution of sPsel and VWF levels during the first week after admission to the ICU, stratified by outcome, is presented in Figure 1. The sPsel and VWF levels were mostly above the upper limit of normal throughout the first week. The admission sPsel and VWF levels were above the upper limit of normal in 63.4% (n = 52) and 85.4% (n = 70) of patients, respectively. No significant differences were found in sPsel levels on each day between survivors and nonsurvivors (Figure 3a). VWF levels were lower during the first 3 days and higher on day 7 in nonsurvivors than in survivors (all *p* < 0.05) (Figure 3b).

## Discussion

The main finding of this study was that both the admission sPsel and VWF levels were lower in patients with than without TIC and were significantly correlated with the coagulation parameter levels. Early VWF levels among trauma patients in the ICU were lower in nonsurvivors than in survivors; however, at the end of the first week, VWF levels became higher in nonsurvivors than in survivors.

In the current study, TIC was defined as an admission PTR of >1.2 according to Frith’s epidemiological study [7], in which a PTR of >1.2 was associated with a stepwise increase in mortality and blood product requirements. In addition, the authors were able to demonstrate that the often-cited coagulopathy threshold of PTR/INR > 1.5 fails to detect 16% of patients with poor outcomes. Using a threshold of PTR > 1.2, 45.1% of the included patients were defined as having TIC in our study and were associated with a higher ISS and 30-day mortality rate. Meanwhile, we selected PC and FVII as the markers of coagulation; these parameters have been described as sensitive indices for coagulopathy and were associated with the outcome of trauma patients [8,9].

VWF is synthesized in endothelial cells and megakaryocytes and is released after stimulation by thrombin or other mediators of thrombosis and inflammation [10]. Activated platelets may occasionally contribute to the circulating pool of VWF in patients with thrombotic disorders, but platelet VWF after release from α granules tends to remain bound to the platelet surface. Thus, we may assume that the majority of plasma VWF comes from endothelial cells and that an increase in plasma VWF levels can indicate endothelial activation/damage. P-selectin is an adhesion molecule present in platelets; it is expressed on the platelet surface upon activation as well as on the endothelium in the presence of an atherosclerotic plaque. However, sPsel in the plasma is thought to arise predominantly from platelets with a minimal contribution from endothelial cells, suggesting that sPsel is likely to reflect platelet activation [11,12]. Increased levels of sPsel/VWF have been observed in patients with stroke, coronary artery disease, acute myocardial infarction, and deep vein thrombosis [13-19]. Endothelial damage and platelet dysfunction also seem to be present in patients with severe trauma and TIC, but the mechanisms by which this damage and dysfunction develop are still largely unknown. Meanwhile, the evolution of sPsel and VWF levels following severe trauma has been poorly described. In our study, the sPsel and VWF levels in trauma patients during the first week after entering the ICU were generally above the normal reference range regardless of outcome, which suggests persistent activation of endothelial cells and platelets in these trauma patients.

In our study, the admission VWF levels in patients with coagulopathy and in nonsurvivors were lower than those in patients without coagulopathy and survivors. The admission VWF levels were also correlated with the admission PC/FVII levels, implicating that early low VWF levels might be mainly attributed to coagulopathy and predict a poor outcome. It is known that after activation by thrombin (mediated by protease-activated receptor), endothelial cells release VWF in approximately 30 min [20]; however, in TIC, thrombin generation is impaired and/or diluted [1]. This causal relationship may explain the lower admission VWF levels in patients with than without coagulopathy. Our VWF results were close to those of a previous study by Ostrowski et al. [21] on biomarker profiles in acute TIC, but conflicted with those of a study by Oliveira et al. [22] in which VWF levels of patients with severe traumatic brain injury were higher in nonsurvivors than in survivors upon admission, at 24 h, and at 7 days. Although the morbidity of coagulopathy in Oliveira’s study is unknown, we infer that the inconsistent results of the two studies may be partly attributed to different morbidity of coagulopathy between cerebral and extracerebral injuries.

During the first week after admission, VWF levels in nonsurvivors peaked on day 7 and were higher than those in survivors. Similarly, the VWF levels of patients with traumatic brain injury in two previous studies [22,23] peaked on day 7 and within the second week after entering the ICU. These findings suggest that observing changes in VWF levels may be helpful in predicting the mortality of trauma patients in the ICU.

Platelet dysfunction after trauma has been characterized in some recent studies [24,25]. Our study also found that although the sPsel levels of trauma patients during the first week were generally above the normal reference range, the admission sPsel levels in patients with coagulopathy were lower than the levels in patients without coagulopathy; they were also correlated with the admission PC/FVII levels. However, no significant differences in the sPsel levels between survivors and nonsurvivors were found during the first week. This may be attributed to the dual nature of severe injury, which promotes both platelet activation and coagulopathy that impedes platelet activation. Our data may partly explain why platelet transfusion has not seemed essential for correction of TIC in numerous clinical trials [26-29]. However, transfusion of normally functioning platelets may be associated with additional benefits such as restoration of the endothelium and modulation of infective and inflammatory sequelae [4].

This study has several limitations. Since initial samples were collected after admission to the ICU, the impacts of emergency treatments prior to admission on coagulation markers had not been evaluated, for example, patients with traumatic brain injury in our study received mannitol to reduce acutely raised intracranial pressure until more definitive treatment can be applied, limited literatures have reported the functions of mannitol on inhibiting platelet activation and increasing vasopermeability of endothelial cells [30,31], its possible effects on sPsel/VWF levels might have interfered our results. Second, as an observational study, it did not allow for independent estimation of the cause-and-effect relationship between sPsel/VWF levels and outcome. Third, because of the limited number of patients and amount of clinical data included, a more detailed classification and analysis of the enrolled patients (e.g., according to the presence of sepsis or organ failure) could not be performed. Fourth, conditions possibly affecting sPsel and VWF levels in the enrolled patients, such as von Willebrand disease or congenital platelet function defects, were not excluded.

In conclusion, our study revealed that sPsel and VWF levels in trauma patients are generally above the normal reference range for at least 1 week. The admission levels of sPsel and VWF are greatly influenced by the presence of coagulopathy such that the relatively lower levels of these parameters in trauma patients often do not predict less platelet/endothelial dysfunction or a better outcome. Moreover, restoration of endothelial function seems to be a very important factor in improving the prognosis of trauma patients. Further prospective studies with larger sample sizes are needed to confirm these conclusions.

## Competing interests

The authors declare that they have no competing interests.

## Authors’ contributions

NT conceived of and designed the study, performed the statistical analysis, and drafted the manuscript. SY collected and analyzed the clinical information of the objectives. ZS participated in the study coordination and helped to draft the manuscript. YP participated in the study designed and carried out the coagulation tests. All authors read and approved the final manuscript.
